# A systematic review of Perinatal Antibiotic Stewardship – where we are, where to go?

**DOI:** 10.1038/s41372-025-02209-0

**Published:** 2025-01-20

**Authors:** Cristina Winteler, Sara Ardabili, Markus Hodel, Martin Stocker

**Affiliations:** 1 Faculty of Health Science and Medicine, University of Lucerne, Lucerne, Switzerland; 2 Department of Gynecology and Obstetrics, Luzerner Kantonsspital LUKS, Lucerne, Switzerland; 3 Department of Neonatology and Pediatric Intensive Care, Children’s Hospital of Central Switzerland, Luzerner Kantonsspital LUKS, Lucerne, Switzerland

**Keywords:** Antimicrobial therapy, Paediatrics

## Abstract

The perinatal period is associated with high antibiotic exposure, which raises concerns about antimicrobial resistance (AMR) and future health impacts. The aim of this comprehensive systematic review, including publications from 2000 to 2022, is to describe the current evidence and state of antimicrobial stewardship (AMS) in the perinatal period and to identify gaps in knowledge for future research. The review included 36 studies from the Americas, Europe, Asia and Australia, involving a total of 64,798 pregnant women and 84,137 newborns. 33 out of 36 studies reported reduced antibiotic use, suggesting the potential to reduce antibiotic exposure. There is a lack of studies in the antepartum and intrapartum periods, of comprehensive AMS strategies across the entire perinatal period, and from low- and middle-income countries with a high burden of maternal and neonatal morbidity and mortality. Future research should include prospective, adequately powered studies including safety endpoints, clinical outcomes and AMR reports.

## Introduction

According to the World Health Organization, the perinatal period is defined as the period between the completed 22^nd^ week of pregnancy and the first seven days after birth. Within the perinatal period, three distinct phases with specific health care priorities can be defined: antepartum, intrapartum and postpartum phase. An optimal start at the beginning of life has a significant impact on a person’s health and well-being [1, 2]. Use of antibiotics in the perinatal period is high with potential impact on antimicrobial resistance (AMR) and future health [3, 4]. AMR is one of the main challenges of medicine with currently more than 1.2 million deaths annually directly related [5, 6]. The perturbation of the development of the non-resilient microbiome in early life plays a key role for future health [7, 8]. Exposure to antibiotics in the perinatal period was reported to be associated with asthma, allergies, atopic dermatitis, obesity, celiac disease, diabetes and other immune disorders later in life [4, 8–11]. In addition, late onset sepsis and necrotizing enterocolitis (NEC) were reported as short-term adverse outcomes in preterm infants treated with prolonged exposure to antibiotics [10, 12–14]. Late onset sepsis and necrotizing enterocolitis are associated with impaired neurological long-term outcomes [15, 16].

Antibiotics are among the most frequently prescribed medications during pregnancy and it is estimated that, in approximately 40% of all pregnancies antibiotic treatments are used [4, 17–20]. The reasons for antibiotic treatment are variable and range from urogenital infections, suspected chorioamnionitis to the prophylactic therapy in cases of Group B streptococci (GBS) carriage [4]. Suspected chorioamnionitis, GBS prevention, prophylactic therapy in case of premature rupture of membranes (PROM) and prophylactic treatment in case of a caesarean section are the main reasons for intrapartum antibiotic administration affecting around two out of three pregnancies [21]. Within the first week of life, fear of early onset sepsis (EOS) is a key driver of antibiotic use [3]. But, the diagnosis of neonatal sepsis is challenging and there is still no accepted standardized definition [9, 21–23]. In case of true EOS, early start of antibiotic therapy is mandatory for optimal outcomes [9, 12, 13, 24–28]. The lack of predictive precision in current diagnostic tools and the need to start treatment early in cases of EOS lead to overtreatment: Up to 15% of all newborns and more than 75% of premature infants with a birth weight below 1500 g receive empirical antibiotics for suspected sepsis. In a recently published study comparing the burden of earl-onset sepsis versus the burden of antibiotic treatment, for one case of culture-proven sepsis, 58 newborns received antibiotics, and 273 antibiotic days were administered [29]. This, together with large variations between hospitals and countries, indicates that there is an enormous potential to safely reduce antibiotic exposure at the beginning of life and thereby reduce the threat of antimicrobial resistance and perturbations of the microbiome [3, 26, 29–32]. We hypothesize that there exists a knowledge gap regarding the efficiency of AMS interventions with lack of consideration of the perinatal period as a whole. We assume that most of the existing studies are of insufficient quality or inadequate size to prove safety of an approach. The aim of this review is to describe the current evidence and state of AMS during the perinatal period and to highlight knowledge gaps for future research.

## Methods

**Fig. 1:**
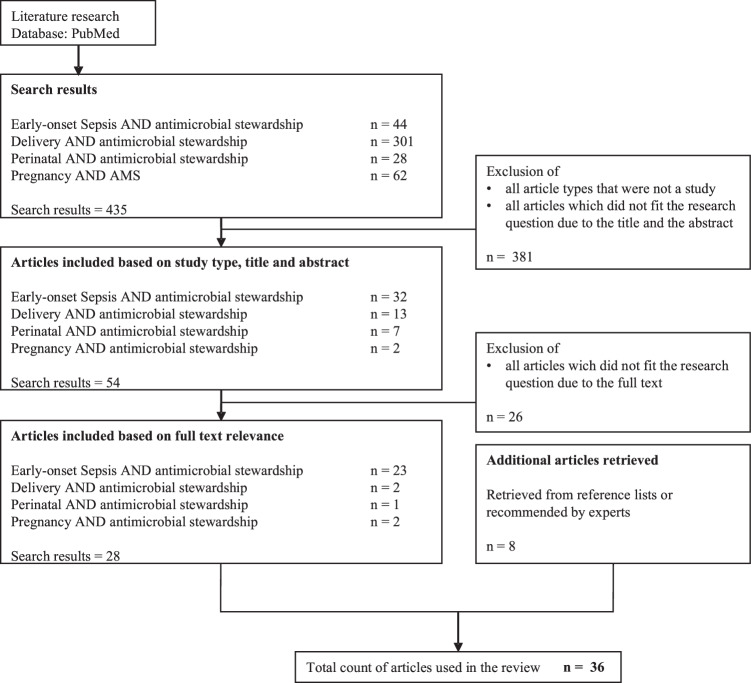
Flowchart of publication search.

For this systematic review, we followed the requirements of the PRISMA checklist for data collection, analysis and reporting (amendment 1). The following four search strings were developed to search for suitable literature in the PubMed database between September 2022 and January 2023: “pregnancy AND antimicrobial stewardship”, “delivery AND antimicrobial stewardship”, “early-onset sepsis AND antimicrobial stewardship” and “perinatal AND antimicrobial stewardship”. The following filters were applied: Literature from 2000 to December 2022, English and German language, available fulltext or abstract. All article types not corresponding to a clinical study (for example reviews, perspectives, comments) were excluded. In a second step, articles which were retrieved from reference lists or were recommended by experts were added. Study selection was done by two authors independently (CW and MS). The flowchart in Fig. 1 provides an overview of the entire selection process.

For every selected study, the following seven characteristics were extracted, organized and summarized in a table (Table 1): Publication year and location, study design, bias assessment, number of participants, AMS intervention, outcome, and safety endpoints/adverse events. The publication year and location were chosen to observe a potential trend in AMS research activity within the last two decades and to describe the most active regions internationally. Within the study method we categorized all prospective randomized controlled trials or prospective quality improvement studies as high-quality, and all retrospective descriptive studies as low-quality study designs. The bias assessment was used to further describe the quality of the study and was done in the form of a separate risk of bias assessment table. Individual AMS interventions were described and, where possible, categorized according to the WHO definitions of AMS interventions (clinician education, patient and public education, institution-specific guidelines, cumulative antibiograms, prior authorization for restricted antimicrobials, de-labeling of spurious antibiotic allergies, prospective audit and feedback, antibiotic timeouts, antibiotic dose optimization, antibiotic duration), while AMS programs were summarized as multifaceted interventions. Interventions regarding diagnostics of suspected infections or empiric start of antibiotics were categorized into guidelines [33]. To analyze the results, we decided to represent the studies regarding their number of participants in two categories: Less than 1000 participants and more than 1000 participants. The threshold of 1000 was chosen pragmatically: Proven infections in the perinatal period are rare and therefore the studied population needs to be sufficient large to get potential generalizable results. The outcome of all studies was analyzed regarding the effect on antibiotic use. To assess safety, the studies were analyzed for the presence of an appropriate powered safety parameter and the incidence of adverse events, mortality or primarily missed sepsis cases with a delayed start of antibiotics. If any of the information were not available, we marked the variable as unknown.

**Table 1:** Overview of publications used in the review.

First author	Year & location	Design	Bias*	Population size	Intervention	Outcome	Safety
**Antepartum**							
Kenyon	2001 Europe	Prospective RCT	1 out of 6	4’809 women	**Guideline:** Antibiotic treatment (erythromycin, co-amoxicillin or both vs placebo) in pregnancies with pPROM	Erythromycin with a significant benefit over placebo for a neonatal composite outcome (death, CLD, neurological impairment), co-amoxicillin with increased NEC rate	Powered for safety outcome Increased rate of NEC with co-amoxicillin
Ya-Zheng Zhao	2022 Asia	Retrospective	Not applicable	Unknown	**Guideline:** Implementation of five rules regarding general prescriptions of antibiotics in pregnancy	Overall significant reduction of antibiotic use	Not powered for safety outcome No report about adverse events
Manju	2022 Asia	Prospective QI-study	4 out of 6	2’068 women	**Guideline:** SAP in low-risk patients for elective surgeries during pregnancy and caesarean sections	Significant increase of single dose SAP-rate with no increase in surgical site infection rate	Powered for safety No adverse outcomes observed
**Intrapartum**							
Witt	2011 Europe	Prospective	3 out of 6	1’112 women	**Guideline:** SAP before skin incision vs after umbilical cord clamping vs placebo in cesarean section	Significantly lower incidence of surgical site infections with antibiotic prophylaxis versus placebo	Powered for safety outcome No adverse outcomes observed
Sommerstein	2020 Europe	Prospective	4 out of 6	55’901 women	**Guideline:** SAP in cesarean section after umbilical cord clamping instead of prior to incision	No significant increase in surgical site infection	Powered for safety outcome No adverse outcomes observed
Sharma	2021 Asia	Prospective QI- study	4 out of 6	342 women	**Guideline:** Restricting antibiotic prophylaxis for uncomplicated births	Significant reduction of intrapartum antibiotic prophylaxis	Not powered for safety outcome 13 sepsis cases reported
Fullston	2019 Europe	Retrospective	Not applicable	200 women	**Guideline:** GBS testing with PCR in term woman with PROM > 18 h and without other risk factors for EOGBS	Significant reduction of intrapartum antibiotic prophylaxis	Not powered for safety outcome No adverse outcomes observed
Hartvigsen	2022 Europe	Prospective QI- study	5 out of 6	366 women	**Guideline:** GBS testing with PCR in laboring woman fulfilling at least one risk factor for EOS with GBS	Significant reduction of intrapartum antibiotic prophylaxis	Not powered for safety outcome No adverse outcomes observed
**Postpartum**							
Money	2017 North-America	Retrospective	Not applicable	362 infants	**Guideline:** Kaiser Sepsis Calculator in well-appearing term infants to mothers with chorioamnionitis	Significant reduction of antibiotic use	Powered for safety outcome One missed sepsis case
Warren	2017 North-America	Retrospective	Not applicable	205 infants	**Guideline:** Kaiser Sepsis Calculator in infants GA ≥34 weeks who received antibiotics at birth	Significant reduction of antibiotic use	Not powered for safety outcome No adverse events observed
Dhudasia	2018 North-America	Retrospective	Not applicable	11’782 infants	**Guideline:** Kaiser Sepsis Calculator in infants GA ≥36 weeks and empirical antibiotics	Significant reduction of antibiotic use Significant reduction of blood testing	Not powered for safety outcome No adverse events observed
Achten	2018 Europe	Prospective	4 out of 6	308 infants	**Guideline:** Kaiser Sepsis Calculator as addition to protocol in children born GA ≥35 weeks with suspected EOS	Significant reduction of antibiotic use	Not powered for safety outcome No adverse events observed
Eason	2019 Europe	Retrospective QI – study	Not applicable	595 infants	**Guideline:** Kaiser Sepsis Calculator in well-appearing term infants with EOS-risk factors	Significant reduction of antibiotic use	Powered for safety outcome No adverse outcomes observed
Achten	2020 Europe	Retrospective	Not applicable	1’708 infants	**Guideline:** Kaiser Sepsis Calculator in infants GA ≥35 weeks and a maternal risk factor or clinical sign of EOS	Significant reduction of antibiotic use Significant reduction of blood testing	Not powered for safety outcome No report about adverse events
Morris	2020 Europe	Retrospective	Not applicable	70 infants	**Guideline:** Kaiser Sepsis Calculator in infants GA ≥34 weeks with proven EOS and at least five days of antibiotics	Significant reduction of antibiotic use	Not powered for safety outcome Increased rate of delayed treatment
Laccetta	2021 Europe	Retrospective	Not applicable	265 infants	**Guideline:** Kaiser Sepsis Calculator in infants GA ≥34 weeks with a risk factor or clinical signs for EOS	Significant increase in number of infants receiving antibiotics by 9% compared to using local guidelines	Not powered for safety outcome One missed case of culture neg sepsis
Frymoyer	2020 North-America	Retrospective QI – study	Not applicable	20’394 infants	**Guideline:** Serial clinical examination in well-appearing ≥35 GA old, regardless the risk factors	Significant reduction of antibiotic use Significant reduction of blood testing	Not powered for safety outcome No adverse outcomes observed
Vatne	2020 Europe	Prospective QI- study	4 out of 6	17 242 infants	**Guideline:** Serial clinical examinations for 24 – 48 h in term neonates at risk for EOS	Significant reduction of antibiotic use	Not powered for safety No adverse outcomes observed
Schmitt	2021 Europe	Retrospective	Not applicable	986 infants	**Guideline:** Serial clinical examinations in asymptomatic term infants at risk for EOS	Significant reduction of antibiotic use Significant reduction of blood testing	Not powered for safety outcome No adverse outcomes observed
Capin	2020 North-America	Retrospective	Not applicable	946 infants	**Guideline:** Stratification of risk factors in infants with respiratory distress, antibiotics should only be started if the indication for delivery was fetal and not maternal	Significant reduction of antibiotic use without missed cases of EOS	Powered for safety outcome No adverse outcomes observed
Cantey	2016 North-America	Prospective	4 out of 6	2502 infants	**Duration:** Automatic stop order after 48 h or stop after 5days when pneumonia or culture negative sepsis	Significant reduction of antibiotic use	Not powered for safety outcome No adverse events observed
Tolia	2017 North-America	Retrospective	Not applicable	674 infants	**Duration:** Automatic stop order after 48 h of antibiotics in VLBW infants	Significant reduction of antibiotic use	Not powered for safety outcome No adverse outcomes observed
Astorga	2018 North-America	Retrospective QI – study	Not applicable	1203 infants	**Duration:** Automatic stop order after 48 h, proactive override possible when clinical sepsis was presumed	Significant reduction of antibiotic use	Not powered for safety No adverse events observed
Lacaze	2014 North-America	Prospective	4 out of 6	1202 infants	**Duration:** Biomarker-guidance by single measurement of CRP at 18 h of life in newborns <35 weeks GA at risk for EOS	Significant reduction of antibiotic use Significant increase of laboratory testing	Powered for safety outcome 44 missed cases of (presumed) EOS
Stocker	2017 Europe	Prospective RCT	2 out of 6	1710 infants	**Duration:** Procalcitonin-guidance to shorten antibiotic therapy duration	Significant reduction of antibiotic use	Powered for safety outcome No adverse outcomes observed
Ykema	2018 Europe	Retrospective	Not applicable	88 infants	**Duration:** Placental analysis as diagnostic tool for EOS in infants less than 32 weeks of gestation	Significant reduction of antibiotic use	Not powered for safety outcome No adverse events observed
Steinmann	2018 Europe	Retrospective	Not applicable	1567 infants	**Clinician education:** Leadership change from control-driven to an empowering regarding antibiotic use	Significant reduction of antibiotic use	Not powered for safety outcome No adverse events observed
Wang	2020 Asia	Retrospective	Not applicable	9297 infants	**Audit and Feedback:** Infectious disease rounds for individual review of each patient in a group of specialists	Significant reduction of antibiotic use Significant increase of taking blood cultures	Not powered for safety outcome No adverse events observed
Arora	2019 North-America	Retrospective QI – study	Not applicable	539 infants	**Multifaceted Intervention**; Kaiser Sepsis Calculator + 36 h antibiotic time out in infants GA ≥ 34 weeks	Significant reduction of antibiotic use	Not powered for safety outcome No adverse events observed
Meyers	2020 North-America	Prospective QI- study	4 out of 6	Unknown	**Multifaceted Interventions**; PDSA cycles, focusing on addressing gaps in the core elements of AMS-programs	Significant reduction of antibiotic use	Not powered for safety outcome No adverse events observed
Hamdy	2020 North-America	Prospective QI- study	4 out of 6	Unknown	**Multifaceted Interventions;** Implementation of various measures to reduce the Vancomycin prescribing rate	Significant reduction of vancomycin use	Not powered for safety outcome No adverse events observed
Singh	2021 North-America	Retrospective QI-study	Not applicable	1363 infants	**Multifaceted Intervention;** Elimination of routine CRP + automatic stop order for antibiotics at 48 h	Significant reduction of antibiotic use	Not powered for safety outcome No adverse events observed
Berardi	2021 Europe	Retrospective	Not applicable	230 infants	**Multifaceted Intervention**; Implementation of procedures to inform medical staff regarding AS in VLBW neonates	Significant reduction of antibiotic use	Not powered for safety outcome Increase in case fatalities
Stritzke	2022 North-America	Retrospective QI-study	Not applicable	479 infants	**Multifaceted Intervention;** EOS guideline + automatic stop order for antibiotics at 5 d + clinical pharmacist involvement	Significant reduction of antibiotic use	Not powered for safety outcome Increased rate of delayed treatment
Graus	2022 South-America	Prospective QI-study	4 out of 6	858 infants	**Multifaceted Interventions**; Seven PDSA cycles for standardized management when EOS suspected	Significant reduction of antibiotic use	Not powered for safety outcome No adverse events observed
Malviya	2022 Asia	Retrospective	Not applicable	7’562 infants	**Multifaceted Interventions;** Implementation of the broad principles of CDC empowering frontline physicians	Significant reduction of antibiotic use	Not powered for safety outcome No adverse outcomes observed

*Compare bias assessment in Fig. 3.

*RCT* randomized controlled trial, *QI* quality improvement, *GA* gestational age, *VLBW* very low birth weight, *SAP* surgical antibiotic prophylaxis, *CLD* chronic lung disease, *NEC* necrotizing enterocolitis, *PCR* polymerase chain reaction, *pPROM* prolonged premature rupture of membranes, *GBS* group B streptococci, *EOS* early-onset sepsis, *CRP* c-reactive protein, *PCT* procalcitonin, *PDSA cycle* Plan-Do-Study-Act cycle.

All studies were sorted according to the time of their intervention in the perinatal period: Antepartum (prenatal), intrapartum (delivery), and postpartum (postnatal). Antepartum was defined after the completed 22^nd^ week of gestation, intrapartum including all studies describing antibiotic use for delivery (including prophylactic antibiotics for GBS carriage, ROM and caesarean section), and postpartum including all studies within the first week of life. If a study included interventions in more than one phase, the study was categorized to the first phase.

## Results

**Fig. 2:**
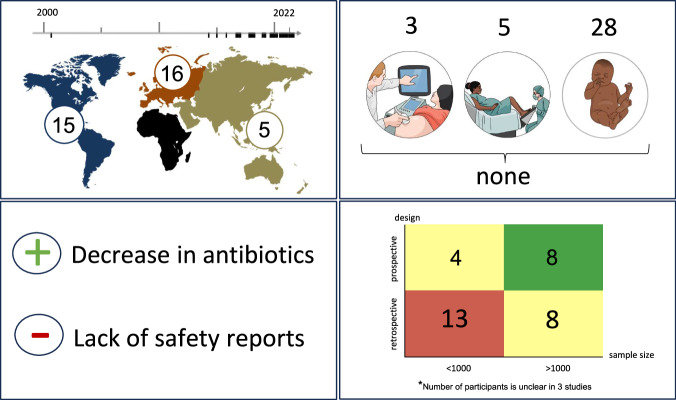
Overview of results.

**Fig. 3:**
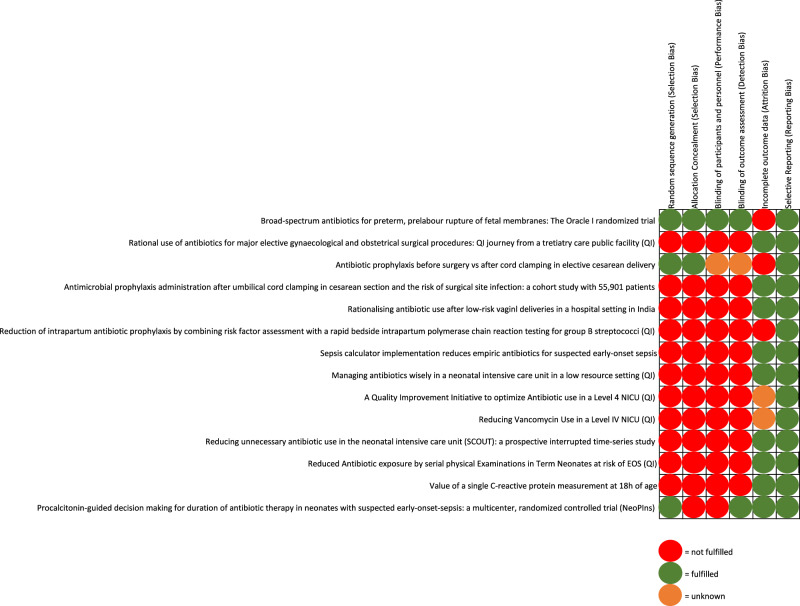
Bias assessment. Bias assessment of all prospective studies (*n* = 14).

We identified 36 studies within the literature research according to the defined criteria (Fig. 1, Table 1). An overview of the most important results is available in Fig. 2. One study was published before 2010 and 34 out of the 36 studies (94%) were published between 2015 and 2022. 16 out of 36 studies originated from Europe, 15 from America and five from Asia and Australia. The study design was retrospective in 22 out of 36 studies (61%). The bias assessment showed in all 14 prospective studies at least one additional bias (Fig. 3). In total, 64’798 pregnant women and 84’137 neonates participated in all studies, whereas the exact number of participants were not clearly stated in three out of 36 studies. 17 of the studies included less than 1000 participants (47%) [34–50]. A total of 15 different interventions were presented across 5 AMS categories: guidelines (*n* = 20), antibiotic duration (*n* = 6), feedback and audit (*n* = 1), clinician education (*n* = 1) and multifaceted interventions (*n* = 8) aimed at reducing the use of antibiotics during pregnancy and the first week of life. The outcome showed a reduction in antibiotic use in 33 out of 36 studies (92%). Nine of the studies were powered for a safety outcome [37, 40, 50–56]. No increase in adverse events was observed in 29 out of 36 studies, while two studies did not report on adverse events or safety. 35 out of 36 studies included interventions in one phase, one in two phases and none in the complete perinatal period.

We identified three out of 36 studies (8%) in the antepartum phase [51, 56, 57]. The largest study in the antepartum phase was a randomized controlled trial investigating use of antibiotics in pregnancies with preterm prenatal rupture of membrane. The study showed a significant reduction (−16.5%) of a compound neonatal outcome (neonatal death, chronic lung disease, cerebral impairment) when erythromycin was given compared to placebo [51]. The use of co-amoxicillin (amoxicillin with clavulanic acid) was associated with an increased rate of neonatal NEC. The other prospective study focused on single dose surgical antimicrobial prophylaxis (SAP) in low-risk patients for elective surgeries during pregnancy and for caesarean section [56]. The result was a significant increase of single dose SAP-rate from 2% to over 60% within 6 months, maintained at 80–90% for more than two years with no increase in surgical site infection rate. The retrospective study reported about five rules regarding general prescriptions of antibiotics in pregnancy and showed a significant reduction in the use of antibiotics [57].

In the intrapartum phase, we identified five out of 36 studies (14%) [34–36, 52, 53]. Two studies focused on surgical antibiotic prophylaxis for cesarean section [52, 53]. One of them analyzed the difference between antibiotic use versus placebo [52], the other the time of application of antibiotic prophylaxis [53]. The study analyzing surgical antibiotic prophylaxis versus placebo showed a lower rate of infectious morbidity in the prophylaxis group [52], while an unchanged risk of surgical site infection was reported when antibiotic prophylaxis was given after cord clamping rather than before incision [53]. A third study monitored the effect of a new guideline restricting antibiotic prophylaxis in uncomplicated births and reported a 75% reduction in antibiotic use [34]. The last two studies looked at the impact of intrapartum polymerase chain reaction testing in GBS-positive mothers and reported a reduction in the need for intrapartum prophylaxis (IAP) by up to two-thirds [35, 36]. No adverse events were observed in four of the five studies, whereas 13 out of 913 infants in the study restricting antibiotic prophylaxis for uncomplicated birth developed sepsis within three days [34–36, 52, 53].

In the postpartum phase, we identified 28 out of 36 studies (78%). With eight out of 28 studies, the Kaiser Permanente Sepsis Calculator is the most analyzed single intervention [37–42, 58, 59]. Seven out of the eight studies were done in a retrospective design. The prospective study analyzing the Kaiser Permanente Sepsis Calculator was a before-after setting with a historical control group [39]. Multifaceted interventions, a combination of different interventions that were implemented at the same time or consecutively, were analyzed in eight studies [43–46, 60–63]. Most of these studies were quality improvement studies and five out of the eight studies had a retrospective design. An automatic stop-order was analyzed in three studies [47, 64, 65]. Two of the three studies were retrospective. The prospective study was an observational study with over 2’500 neonates included. In all three studies, antibiotic prescriptions were stopped automatically after 48 h of treatment. Serial physical examinations were analyzed in three studies [48, 66, 67]. Two of the three studies were retrospective. In all three studies, clinically healthy neonates with risk factors for EOS were observed for 48 h without antibiotic treatment. Additional two studies analyzed a biomarker-guided approach [54, 55]. Both studies had a prospective design with more than 1000 participants. One study analyzed the effect of c-reactive protein-guidance 18 h after start of antibiotic therapy, the other study used a procalcitonin-guided algorithm to shorten antibiotic treatment. The remaining four studies analyzed four different interventions based on placental analysis, leadership style for empowerment, infectious disease rounds and stratification of risk factors [13, 49, 50, 68]. All four studies were done in a retrospective design. In 27 out of the 28 studies in the postpartum phase, the outcome showed a reduction of antibiotic use, measured by various endpoints [13, 34–41, 43–68]. In one study analyzing the Kaiser Permanente Sepsis Calculator, the number of newborns identified for antibiotic therapy increased [42]. Regarding safety, five of the included studies were powered for a safety outcome showing different results [37, 40, 50, 54, 55]. No adverse events were observed in 22 out of the 28 studies, one study did not report about adverse events or safety [59]. In three studies, at least one EOS case was missed during the intervention phase [37, 42, 54]. An increased rate of delayed antibiotic treatment was observed in two studies [41, 45]. One study showed an increase in case fatalities, a re-analysis could not show any association of this result with a delayed or insufficient antibiotic therapy [44].

## Discussion

Within the last decade, literature regarding AMS in the perinatal period increased remarkably. The increase of publication is mainly focused on the postpartum phase. Overall, a reduction of antibiotic use in the perinatal period is possible: In 33 out of the 36 studies analyzed, the introduction of an AMS intervention reduced exposure to antibiotics in the defined study population. On the other hand, this review shows a lack of studies in the ante- and intrapartum phase and a complete gap of AMS analysis including the whole perinatal period. Around two thirds of the studies were done in a retrospective and therefore low-quality design. In addition, most of the studies were not powered to assess the safety of the intervention.

Around half of the studies analyzed were published between 2014 and 2019 and their number doubled from 2020 to 2022, underlining a strong trend. First, the reason for this trend may be grounded in the call of international organizations as the World Health Organization to take global action on AMR to improve antibiotic treatment by increased surveillance and research [5]. Second, there is increasing evidence that unnecessary antibiotics in the perinatal period has an impact on the individual microbiome with potential impact for future health [3, 7]. And third, there is evidence that antibiotic exposure for only 48 h within the first week of life has major effects on the microbiome and AMR gene selection and that these changes are still relevant one year later [8]. The distribution of pathogens causing EOS and resistance patterns have changed over time and increased morbidity and mortality due to AMR is a major concern, particularly in middle- and low-income countries [5, 6]. Therefore, the lack of studies from Africa and Asia reported in this review is a worrying gap in the current knowledge and needs to be addressed in future AMS programs.

When analyzing the study’s design, it is noticeable that around two thirds of the 36 studies included were conducted in a retrospective design. This limits the significance of the results. In addition, around half of the studies had a sample size below 1000 participants. Whereas, a small sample size does not automatically mean low quality, an AMS study reducing antibiotic prescriptions must show a safety endpoint. The inclusion of safety parameters is missing in around a quarter of the studies analyzed, which represents an obstacle to safely introduce the interventions. A non-inferiority analysis reporting missed sepsis cases, delayed antibiotic initiation in culture-proven sepsis, antibiotic restarts due to recurrent infections, and morbidity and mortality are important safety parameters. Culture-proven bacterial infections in the perinatal period are overall relatively rare and power calculations for non-inferiority usually results in a high number of participants. As an example, the prospective, multicenter randomised controlled intervention trial NeoPInS analyzing a procalcitonin-guided algorithm to safely shorten antibiotic therapy in a cohort of more than 1’700 neonates with suspected EOS reported a highly significant result for superiority (reduction of antibiotics), but failed to prove non-inferiority [55]. Nevertheless, this trial together with some other prospective randomized trials published in high-stakes medical journals show the feasibility of large, prospective AMS studies.

Interestingly, 33 out of the 36 included studies reported a reduction of antibiotic prescriptions in the analyzed population. Whereas, we must consider a possible publication bias, it demonstrates that a reduction of antibiotic exposure in this vulnerable phase is feasible. An additional conclusion from this study is that there is overtreatment. Overall, 15 different interventions were used within the 36 analyzed studies. The Kaiser Permanente Sepsis Calculator and multifaceted AMS interventions as quality improvement programs were the most often studied strategies. Because of the large variety of interventions and study designs, it is not possible to conduct a meta-analysis comparing the effectiveness of different interventions.

This review shows important knowledge gaps regarding AMS within pregnancy and deliveries within the last two decades. Only three studies were included in the antepartum phase. One published in 2001 and two in 2022, hopefully indicating a start to fill this gap. This is urgently needed due to the estimation that around 40% of pregnancies are exposed to antibiotics [4]. Assessment tools such as the quick Sequential Organ Failure Assessment score (qSOFA) may help overcome some of the barriers to decision-making about antibiotic prescribing for pregnant women, but high-quality studies are lacking. Future studies are urgently needed to answer the main question about AMS in pregnancy: Which algorithm helps to diagnose bacterial infections in pregnancy with high accuracy and reduce unnecessary empirical antibiotic therapy? On the other hand, ethical concerns for clinical studies in pregnancies potentially increasing the risk to the pregnant women and the unborn child may be a reason for the low number of studies. Within the intrapartum phase, antibiotic prophylaxis for GBS was focused in clinical studies before 2000. The rate of neonatal EOS declined markedly within the last two decades [29, 69]. The strategy of prophylactic antibiotics for GBS positive pregnancies before delivery is probably responsible for a part of the decline, whereas the optimal strategy in the current area remains unknown: While there is probably no safe way to reduce overall prophylactic antibiotic exposure in GBS-positive pregnancies, the question remains, is there a way to safely reduce antibiotic prophylaxis in specific situations? What are the conditions necessary to safely administer surgical antibiotic prophylaxis for caesarean section after cord clamping rather than before incision? To answer these questions, we need to know exactly what effect a single dose of intrapartum antibiotics has on the developing neonatal microbiome and clinical outcomes. And does this effect depend on the type of antibiotic administered? In addition, the pathogen spectrum is changing over time and the resistance rates are increasing, reinforcing the need for new studies, particularly in low- and middle-income countries with a high burden of maternal and neonatal morbidity and mortality due to AMR. Last, it is striking that no study includes the perinatal period as a whole. Whereas the development and rise of perinatal centers internationally indicates an increased understanding of the importance of taking a holistic view of the perinatal period for clinical work, this needs to be further developed in clinical research. Antibiotic exposure during the whole perinatal period may have an impact on the neonate.

The main limitations restricting comparability and conclusions of this review are the low availability of high-quality studies and the large heterogeneity of study designs. Additionally, despite a thorough search of the PubMed database, possible relevant studies in other sources of biomedical and life science literature were not included. And third, because of the definition of the postpartum phase, antibiotic exposure and AMS opportunities in the neonatal intensive care unit beyond the first week of life are not covered. Nevertheless, various knowledge gaps and starting points for future research can be identified based on these findings (Table 2). First, the consideration of the perinatal period as a whole is key to support close communication of all involved clinical disciplines to plan and conduct clinical research improving AMS. Linked medical databases of the mother and the newborn facilitate to analyze the current state and to coordinate future research activities. Second, there is a need for prospective and adequately powered trials with clinical and safety endpoints in all three phases of the perinatal period. This need is most urgent in the antenatal and intrapartum periods. More studies are being published on AMS in the first week of life, but the heterogeneity of the interventions analyzed is high and safety or clinical outcomes are often not reported. In addition to AMR, clinical health outcomes in later life, such as asthma, allergies, atopic dermatitis, obesity, celiac disease, diabetes and other immune disorders, may demonstrate the burden of antibiotic therapy [4, 7–11]. Third, AMS interventions need to be tailored to the local context for implementation. There is most probably not one intervention fitting all context and different interventions need to be tested in various conditions. Therefore, future research must include more ethnically, racially and culturally diverse populations from low- and middle-income countries to reduce the high burden of maternal and neonatal morbidity and mortality. On the other hand, promising techniques for early detection of pathogens and AMR, such as nucleic acid amplification technologies (NAAT) and multiplex polymerase chain reaction (mPCR) need to be further tested in algorithms in high-income settings [35, 36]. For example, the incorporation of mPCR into algorithm-based approaches to electronical clinical records may help to support balanced decision-making on antibiotic therapy in the future [70, 71]. The development of a toolbox of various interventions for different situations may help for further dissemination and implementation of AMS. In the end, the implementation of AMS interventions is always a change process. Health care workers need to have a sense of urgency for AMS before adapting and changing their behavior. Therefore, the increase of the AMR challenge worldwide and the impact of antibiotic therapy on the child’s microbiome with potential impact of their future health are the cornerstones of every AMS program. Knowledge, communication, and education of all involved healthcare workers in the perinatal period are key to redirect the current increasing trends for AMR and chronic health conditions in the worldwide population.

**Table 2:** Summary of important methodological aspects and research questions for future studies in perinatal antimicrobial stewardship.

**Five important methodological aspects for future studies in perinatal antimicrobial stewardship**
**Mother and infant belong together:** Paired data collection of mother and infant by linked medical and research databases including maternal and neonatal antibiotics
**Environment is key:** Consider ethnically, racially and culturally diverse populations including low- and middle-income countries with a high burden of maternal and neonatal morbidity and mortality
**Prospective planning for high-quality research:** Strive for prospective randomized trials or adaptive multi-arm, multi-stage study designs (e.g. platform trials)
**Non-inferiority aspect:** Powered for safety outcomes as missed sepsis cases, delayed start of antibiotics in case of infections, morbidity and mortality (burden of disease)
**Superiority aspect:** Antibiotic use and long-term follow-up or proxy for clinical outcomes and/or antimicrobial resistance rates or microbiome analyses including antimicrobial resistance genes (burden of therapy)
**Five important research questions for future studies in perinatal antimicrobial stewardship**
**Antibiotic use in pregnancy:** What algorithm can accurately assess the risk of suspected maternal infections in pregnancy, and how can we ensure compliance by health care workers in prescribing empiric antibiotic therapy?
**Antibiotic use in neonates:** What algorithm can accurately assess the risk of suspected neonatal bacterial infections, and how can we ensure compliance by health care workers in prescribing empiric antibiotic therapy?
**Antibiotic prophylaxis:** How can we safely reduce perinatal antibiotic prophylaxis in specific situations and what are conditions necessary to safely administer prophylaxis for caesarean section after cord clamping?
**New technologies:** How can we use newer technologies for early detection of pathogens and AMR, or AI and machine learning to support diagnostic accuracy and antibiotic prescription decisions?
**Effect of antibiotics:** What effect has a single dose of intrapartum or postpartum antibiotics on the developing neonatal microbiome and clinical outcomes and does this effect depend on the type of antibiotic administered?

*AMR* Antimicrobial resistance, *AI* artificial intelligence.

## Conclusion

In recent years, published studies regarding AMS in the perinatal period increased remarkably reporting the feasibility and possibility to reduce antibiotic therapy in this vulnerable phase. There is a lack of studies in the ante- and intrapartum phase and a complete gap of AMS analysis including the whole perinatal period. Many of the studies were done in a low-quality design or were not powered to assess the safety of the intervention.

## Data Availability

All the included studies are accessible via Pubmed.
